# A Novel *puf-A* Gene Predicted from Evolutionary Analysis Is Involved in the Development of Eyes and Primordial Germ-Cells

**DOI:** 10.1371/journal.pone.0004980

**Published:** 2009-03-25

**Authors:** Ming-Wei Kuo, Sheng-Hung Wang, Jui-Chin Chang, Chien-Huei Chang, Ling-Jyun Huang, Hsin-Hung Lin, Alice Lin-Tsing Yu, Wen-Hsiung Li, John Yu

**Affiliations:** 1 Institute of Cellular and Organismic Biology, Academia Sinica, Taipei, Taiwan; 2 Genomics Research Center, Academia Sinica, Taipei, Taiwan; 3 Department of Ecology and Evolution, University of Chicago, Chicago, Illinois, United States of America; 4 Biodiversity Research Center, Academia Sinica, Taipei, Taiwan; Ecole Normale Supérieure de Lyon, France

## Abstract

Although the human genome project has been completed for some time, the issue of the number of transcribed genes with identifiable biological functions remains unresolved. We used zebrafish as a model organism to study the functions of Ka/Ks-predicted novel human exons, which were identified from a comparative evolutionary genomics analysis.

In this study, a novel gene, designated as *puf-A*, was cloned and functionally characterized, and its homologs in zebrafish, mouse, and human were identified as one of the three homolog clusters which were consisted of 14 related proteins with Puf repeats. Computer modeling of human Puf-A structure and a pull-down assay for interactions with RNA targets predicted that it was a RNA-binding protein. Specifically, Puf-A contained a special six Puf-repeat domain, which constituted a unique superhelix half doughnut-shaped Puf domain with a topology similar to, but different from the conventional eight-repeat Pumilio domain. *Puf-A* transcripts were uniformly distributed in early embryos, but became restricted primarily to eyes and ovaries at a later stage of development. In mice, *puf-A* expression was detected primarily in retinal ganglion and pigmented cells. Knockdown of *puf-A* in zebrafish embryos resulted in microphthalmia, a small head, and abnormal primordial germ-cell (PGC) migration. The latter was confirmed by microinjecting into embryos *puf-A* siRNA containing *nanos* 3′ UTR that expressed in PGC only. The importance of Puf-A in the maturation of germline stem cells was also implicated by its unique expression in the most primitive follicles (stage I) in adult ovaries, followed by a sharp decline of expression in later stages of folliculogenesis. Taken together, our study shows that *puf-A* plays an important role not only in eye development, but also in PGC migration and the specification of germ cell lineage. These studies represent an exemplary implementation of a unique platform to uncover unknown function(s) of human genes and their roles in development regulation.

## Introduction

Comparing human and mouse/rat genomic sequences, Nekrutenko *et al*. predicted new human protein-coding exons [1]. This approach takes advantage of the fact that in coding regions, synonymous substitutions occur much more frequently than non-synonymous ones. They predicted 13,711 novel exons that were present in both the rodent and human genomes, but the predicted transcripts remained to be validated and their biological functions remained to be demonstrated [2], [3]. When we started this study, 4,768 of the originally predicted new exons were already recognized as genes or pseudogenes, so we used the remaining 8,943 potential novel human exons to search for zebrafish orthologs in a zebrafish database (http://www.sanger.ac.uk/Projects/D_rerio/). From this *in silico* analysis, we found 308 potential genes that had yet no defined biological function (unpublished data). In this study, we chose a novel *puf-A* gene from the 308 potential genes to characterize its function in zebrafish, mouse, and human.

Zebrafish (*Danio rerio*) has become a favorite vertebrate model for genetic and developmental studies due to its attributes such as a small body size, rapid development, frequent reproductive cycles (1∼2 weeks), short maturation period (3 months), large-scale genetic screening, and easy maintenance [4]. In addition, zebrafish mutations are usually faithful phenocopies of many human disorders [5].

The Puf family is an evolutionarily conserved protein family named after Pumilio (*Drosophila*) and FBF (Fem-3 mRNA-binding Factor, *Caenorhabditis elegans*). Puf proteins have been found in various organisms, including yeast, *C. elegans*, *Drosophila*, zebrafish, *Xenopus*, mouse, and human, but their function is largely unclear. The first Puf protein, Pumilio, identified from *Drosophila*, was known to repress translation of *hunchback* mRNA in the posterior half of the *Drosophila* embryo, thereby permitting abdominal development [6]. In addition to its role in posterior patterning of embryos, *Drosophila* Pumilio functions in the development of germline stem cells [7]. Puf family members are usually identified by the presence of eight tandem Puf repeats of ∼35–39 amino acids [8] and the repeat binds to specific sequences in the 3′ untranslated region (UTR) of a target mRNA.

In this study, we conducted various experiments to show that a novel *puf-A* gene is involved in eye and primordial germ-cell (PGC) development. Using the SMART server, we identified 14 *puf-A*-related proteins in human, mouse and zebrafish. We studied their phylogenetic relationships of these 14 proteins. Moreover, a computer modeling of human Puf-A predicted that it is a unique RNA-binding protein composed of six Puf repeats.

## Results

### Expression and cDNA cloning of the novel *puf-A* gene in zebrafish

**Figure 1:**
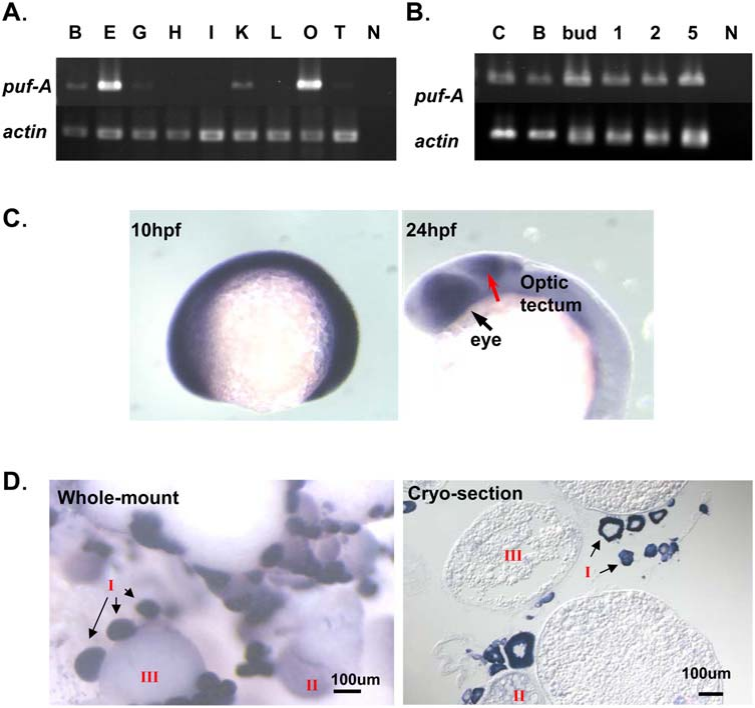
Expression of *puf-A* in zebrafish using RT-PCR and *in situ* hybridization. (A) Gene expression in adult tissues of zebrafish was analyzed by RT-PCR and electrophoresis with *puf-A* primers (upper panel) or actin primers (lower panel, as the internal control). Notation: B, brain; E, eye; G, gill; H, heart; I, intestine; K, head kidney; L, liver; O, ovary; T, testis; N, negative control. (B) The *puf-A* gene was expressed in various stages of zebrafish embryo. C, cleavage; B, blastula; 1, 1 day post-fertilization (dpf); 2, 2 dpf; 5, 5 dpf; N, negative control. (C) Whole-mount *in situ* hybridization with *puf-A* antisense riboprobe on zebrafish embryo. The *puf-A* expression at 10 h post-fertilization (hpf) (tailbud stage) and 24 hpf (25-somite stage) with lateral overview. The black arrow points to the eye and the red arrow to the optic tectum. (D) Whole-mount and cryo-section *in situ* hybridization with a *puf-A* antisense riboprobe in adult ovaries. In adult ovaries, a staging series of oocyte development was characterized by the diameter of various oocytes [9], [10]. Stage I, primary growth follicles (<0.1 mm); stage II, previtellogenic (0.1–0.30 mm); and stage III, vitellogenic (>0.30 mm).

In zebrafish, the *puf-A* gene was found to express at a high level by RT-PCR in the eyes and ovaries, and to a lesser degree in the brain, head kidney (pronephros), and testes (Fig. 1A). Moreover, this gene was found to express at all stages of zebrafish embryo development (Fig. 1B). *In situ* hybridization confirmed that *puf-A* was ubiquitously expressed in zebrafish embryos from fertilization to early somitogenesis, but at a later stage of embryo development, its expression was restricted primarily to the eyes and optic tectum (Fig. 1C).

In addition, in adult zebrafish ovaries, *puf-A* was found to be prominently expressed in early immature follicles that were small in size and nested with other developing oocytes (Fig. 1D). In general, the process of folliculogenesis could be divided into five stages, beginning with the early germline stage I (primary growth) cells that appeared in clusters, through the pre-vitellogenic stage II and the vitellogenic stage III, and ending with the mature or ovulated stages IV and V [9], [10]. As shown in Fig. 1D, strong *puf-A* mRNA expression was noted in primitive stage I ovarian follicles that appeared spherical in shape with diameter less than 100 µm, nesting with other developing . But this expression declined sharply and became negligible in subsequent stages of oocyte development (e.g. stages II and III). The results of *in situ* hybridization of ovary cross-sections confirmed that *puf-A* mRNA expressed prominently in the cytoplasm of stage I follicles which appeared in clusters. In contrast, stage II and III ovarian follicles showed no discernible expression of the *puf-A* transcript (Fig. 1D). It seems that the expression of *puf-A* occurs when the first wave of follicles begin their process of folliculogenesis.

**Figure 2:**
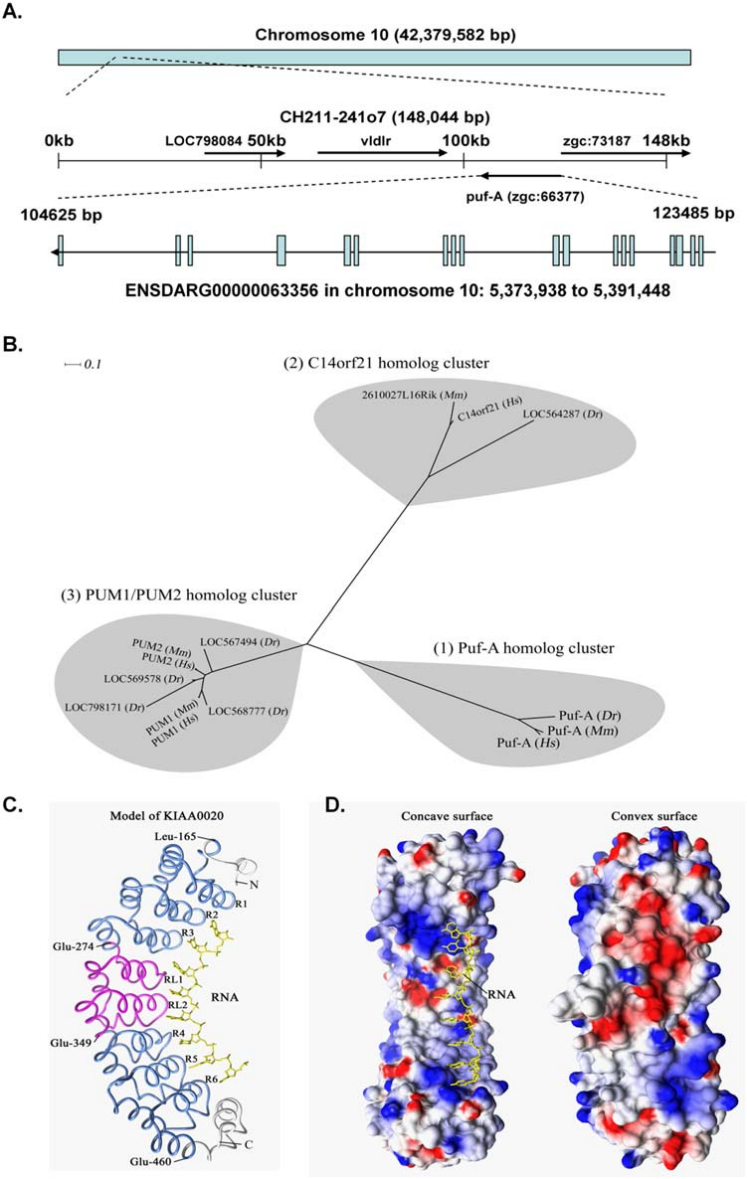
Genetic map of the *puf-A* locus of zebrafish, the unrooted phylogenetic tree, computer modeling of human Puf-A and its electrostatic surface representation. (A) Genetic map of the zebrafish *puf-A* locus and the exon/intron structure of the *puf-A* transcript were constructed through blasting the *puf-A* cDNA sequence to the genome databases of the NCBI and Ensembl websites. The puf-A (zgc: 66377) is ENSDARG00000063356 in chromosome 10: 5,373,938 to 5,391,448 (Ensembl 44). (B) The unrooted phylogenetic tree of human (*Hs*), mouse (*Mm*) and zebrafish (*Dr*) Puf proteins. Phylogenetic analysis was performed using the PHYLIP 3.67 package as described in Method. These Puf proteins could be grouped into three clusters: (1) the Puf-A cluster, (2) the C14orf21 cluster, and (3) the PUM1/PUM2 homolog cluster. (C) Modeling the Puf domain of human Puf-A. This model, built by MODELLER 9v3 as described in Methods, represents the corresponding Puf domain for binding with RNA (yellow). This Puf domain of Puf-A contains six Puf repeats distributed in two regions (R1 to 3 and R4 to 6; blue), and each region contains three repeats. The magenta color refers to the middle region of the Puf domain. The N and C terminal ends of this Puf domain are indicated. (D) Electrostatic surface representation of the Puf domain. The electrostatic potentials were calculated by DELPHI as described. The left panel shows the areas on the concave surface with positive potentials (blue) which interacts with RNA (yellow). The right panel represents the convex surface, where the negative potentials are shown mainly as the acidic (red) and a few hydrophobic (white) areas.

A full-length *puf-A* cDNA was cloned from RNA of zebrafish ovaries (Fig. S1). It is 2,053 bp in length and contains an open reading frame of 629 amino acids (Fig. S1). Further blasting in the NCBI and Ensembl websites demonstrated that the BAC clone #CH211-241o7 contained the full-length *puf-A* gene [ENSDARG00000063356 (Ensembl 44)] with 18 exons on a region of ∼18.8 kb in chromosome 10: 5,373, 938 to 5,391,448 (Fig. 2A). The cDNA/protein sequence of zebrafish Puf-A corresponds to protein LOC394185 (zgc: 66377) in the NCBI with gene ID of 394185 (accession # of protein sequences, XP_695580.2, Table 1) with an exception of five amino acid residues. At residues 558∼562, the sequence is “Glu-Arg-Phe-Ser-Arg” in Puf-A from our study (Fig. S1), but “Gly-Lys-Tyr-Lys-Met” in LOC394185 (Table 1); and this discrepancy arises from a difference in lengths of exons 16 and 17.

**Table 1:** Information on the putative Puf proteins in humans, mice, and zebrafish.

Species	Puf proteins	Gene ID a	Gene location a	Accession #s of protein sequences	Length (amino acids)	Number of Puf repeats d
**Human**	Puf-A (KIAA0020)	9933	9p24.2	Q15397 b	648	6
	C14orf21	161424	14q12	Q86U38 b	636	7
	Pumilio (PUM1)	9698	1p35.2	Q14671 b	1,186	8
	PUM2	23369	2p22-p21	Q8TB72 b	1,066	8
**Mouse**	Puf-A (D19Bwg1357e)	52874	19	Q8BKS9 b	647	6
	2610027L16Rik	67842	14	Q8BMC4 b	636	5
	Pumilio (PUM1)	80912	4	Q80U78 b	1,189	8
	PUM2	80913	12	Q80U58 b	1,066	8
**Zebrafish**	Puf-A e	394185	10	XP_695580.2 c	629	6
	LOC564287	564287	3	XP_692728.2 c	604	5
	LOC568777	568777	16	XP_697221.2 c	457	4
	LOC567494	567494	13	NP_001096040.1 c	1,106	6
	LOC569578	569578	20	XP_698067.2 c	164	3
	LOC798171	798171	18	XP_001338629.1 c	182	3

a Annotations described in the Entrez Gene database at NCBI.

b Accession number used in Swiss-Prot.

c Accession number used in the RefSeq database.

d The Puf repeats were identified by the SMART server.

e According to the annotations in Entrez Gene database, the old gene symbol for *puf-A* is “zgc:66377” and the name for protein is “hypothetical protein LOC394185”.

### Phylogenetic relationships of Puf-A-related proteins

A search for Puf-A-related sequence fragments in the databases suggested that the Puf-A in zebrafish is a member of the Puf family. Each Puf protein contains a Puf domain that consists of several tandem Puf repeats of 36 amino acids [11], [12]; the Puf domain has also been known as the pumilio homolog domain [12]. In total, we identified 14 Puf-related proteins of zebrafish, mouse, and human using the SMART server (Table 1 and Fig. 2B). A phylogenetic tree constructed using the PHYLIP package suggests that these 14 Puf proteins can be grouped into three clusters: (1) the Puf-A homolog cluster, (2) the C14orf21 homolog cluster, and (3) the PUM1/PUM2 homolog cluster (Fig. 2B).

Sequence similarities among these Puf proteins in each cluster were analyzed and categorized (Fig. 2B). In this study, the human and mouse Puf-A homologs, i.e., KIAA0020 and D19Bwg1357e in Table 1, are designated, respectively, as the human and murine Puf-A, respectively. BLASTP analysis revealed that human Puf-A shared 89% identity in the aligned 647 amino acid residues with murine Puf-A and 66% identity with zebrafish Puf-A in the aligned 621 residues. However, compared with human related proteins in the other two categories, human Puf-A and human C14orf21 showed no significant similarity and human Puf-A and Pumilio (PUM1) shared only 21% identity in the aligned 241 amino acid residues (Fig. 2B and Table 1). Similarly, human Puf-A and human PUM2 shared only 20% identity in the aligned 240 residues. Thus, members of the Puf-A cluster are similar to each other, but distinct from the members of the other two clusters. Based on the results of the phylogenetic and sequence similarity analyses, Puf-A homologs could be grouped into a single cluster (Fig. 2B).

On the other hand, within the cluster of C14orf21 homologs (Fig. 2B), human C14orf21 showed 84% identity with murine 2610027L16Rik in the aligned 581 residues and 34% identity with zebrafish LOC564287 in the aligned 619 residues. However, human C14orf21 showed no significant similarity with human Pumilio (PUM1), and human C14orf21 and human PUM2 shared only 23% identity in the aligned 140 amino acid residues. Thus, C14orf21 homologs could be grouped into a single cluster (Fig. 2B).

As to the cluster of PUM1/PUM2 homologs (Fig. 2B), human Pumilio (PUM1) and PUM2 shared 75% identity in the aligned 1,076 residues and human and mouse Pumilio (PUM1) shared 98% identity in the aligned 1,189 residues. In zebrafish, there were four Puf proteins in this cluster (Fig. 2B and Table 1) and their similarity analyses were described in Data S1. The results described above support the clustering of the homologs of PUM1 and PUM2 into one group in our phylogenetic tree analysis (Fig. 2B). The multiple sequence alignments of these 14 Puf proteins were shown in Fig. S2.

### Computer modeling of human Puf-A

Based on the crystal 3D structure of the human Pumilio domain with 1.9 A resolution [13], we conducted a computer modeling of the Puf-domain for human Puf-A (KIAA0020) using its 336 amino acid residues from Asp-151 to Ile-486 (Fig. 2C). The quality of this modeling evaluated by the VADAR server showed that 100% of the residues were in the allowed regions of the Ramachandran diagram [14].

The computer model of human Puf-A predicted its structure to be composed of six Puf repeats, each of which constitutes a unique superhelix, half doughnut-shaped Puf domain (Fig. 2C). The six Puf repeats are distributed in two separate regions from Leu-165 to Glu-273, and from Ala-350 to Glu-460 (R1 to 3 and R4 to 6, respectively; shown in blue in Fig. 2C). These six repeats are structurally aligned with corresponding repeats of the template used in this computer modeling. Moreover, each repeat has three helices and the second helix, which is located at the inner, concave face of the model, and interacts with RNA (yellow in Fig. 2C), exhibiting characteristic features of a conventional Puf repeat [13].

On the other hand, the sequence from Glu-274 to Glu-349, which represents the middle region of this model (RL1 and RL2; shown in magenta in Fig. 2C), contains no typical Puf repeats identifiable by the SMART server. Detailed analysis of this model showed that this middle region possesses a length of segment close to two tandem Puf repeats (76 residues) and each of these “repeat-like” structures exhibits features of three-helix similar to a typical Puf repeat. It is concluded that this middle region mimics two Puf repeats structurally. Thus, the overall structure of Puf-A features a six-Puf-repeat domain with an intermediate region of two repeat-like segments so that it displays a topology similar to the conventional eight-repeat Pumilio homolog domain [13]. Furthermore, this computer model of human Puf-A predicts that it is a new RNA-binding protein, distinctly different from the Pumilio domain.

In addition, the values of electrostatic potentials on the molecular surface of this model of human Puf-A were calculated. As shown in Fig. 2D, an asymmetric distribution of electrostatic potentials was noted for the Puf domain of Puf-A: its concave surface has predominately positive basic electrostatic potentials (shown in blue; left panel of Fig. 2D), presumably for RNA binding, while the convex surface in this model is acidic (red) with partly hydrophobic (white) areas (right panel). Similar properties of the electrostatic surface of this model were also observed in the crystal structure of Pumilio [13].

### Eye defects in the MO knockdown of the *puf-A* gene

**Figure 3:**
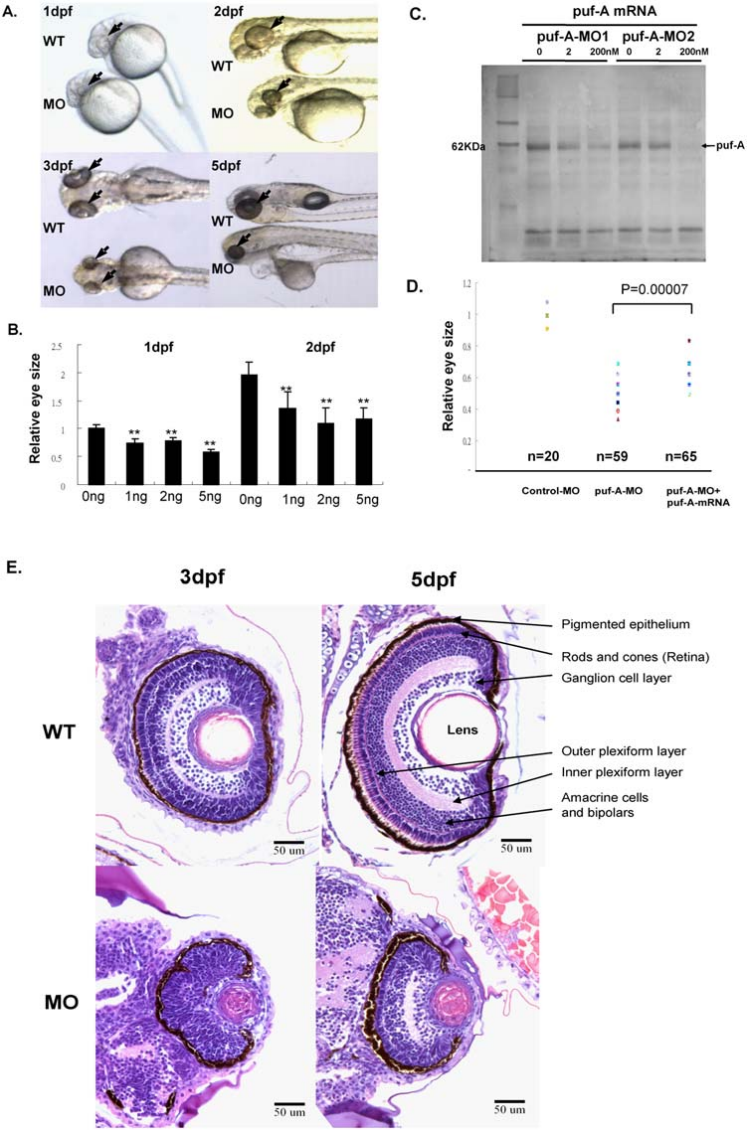
The phenotypes of *puf-A* morphants in the zebrafish. (A) Zebrafish embryos at the 1∼4-cell stage were treated with 5 ng *puf-A* morpholino (MO1) by microinjection. The phenotypes of the wild-type and morphants are shown in lateral view at 1, 2, 3, and 5 days post-fertilization (dpf) after treatment. Black arrows point to the eyes. (B) Various amounts of MO1 were microinjected into zebrafish embryos, and the eye size was measured at 1 or 2 dpf and compared to the eye size of control fish. The “relative eye size” was defined by the value of eye size in MOs relative to the average size of eyes in normal embryos of WT fish. The average value of eye size in normal embryos at 1dpf was considered as 1. Error bars represent the standard error of the mean. ** refer to *p*<0.01 Student's *t*-test). (C) 0, 2, 200 nM *puf-A*-MO1 or *puf-A*-MO2 were added to the *puf-A* generated through *in vitro* transcription/translation reactions. One microliter of the reaction mixture was separated on 10% SDS/PAGE, blotted, incubated with streptavidin-AP, and developed with NBT-BCIP reagents. (D) The 5 ng control- or *puf-A*-MO1 was used for microinjection. In addition, 200 pg of capped *puf-A* RNA was co-injected with *puf-A*-MO1 to check the specificity of MO knockdown. The “relative eye size” was defined as above. The eye size was measured at 2 dpf. Control-MO, *n* = 20 embryos; *puf-A*-MO1, *n* = 59; *puf-A*-MO1+ capped RNA, *n* = 65. The *p* value in Student's *t*-test for the difference between *puf-A*-MO and *puf-A*-MO+mRNA was <0.00007. (E) Transverse histological sections of zebrafish wild-type and morphant (*puf-A*-MO1) eyes stained with hematoxylin and eosin at 3 and 5 dpf.

To examine the biological function of the *puf-A* gene, zebrafish embryos at the 1∼4 cell stage were injected with one of the two *puf-A*-MO antisense oligonucleotides, MO1 and MO2 (see “Materials and Methods” and Fig. S1 for locations of target). As illustrated in Fig 3A, MO1 morphants clearly showed small eyes, a small head, and brain edema at 1 and 2 dpf. Relative to the eye size of WT fish, there were significant reductions in eye size among the morphants in a dose-responsive manner (∼40% reduction with 5 ng *puf-A*-MO1; Fig. 3B). The *puf-A*-MO2 gave results similar to those of the *puf-A*-MO1.

In order to further demonstrate the efficiency of MO1 and MO2, *in vitro* transcription/translation of *puf-A* was performed in the presence or absence of 0∼200 nM MOs. It was shown that these MOs blocked *puf-A* translation *in vitro*, especially at high concentration (Fig. 3C). The specificity was further confirmed by the experiment in which the addition of capped *puf-A* RNA partially but significantly rescued the phenotype of eye size in MO-induced morphants *in vivo* (*p* value<0.00007; Fig. 3D).

Furthermore, in order to circumvent the potential issue of “off-target effects”of MOs, not only a wide range of MOs (1 to 10 ng/embryos) was used for gene knockdown experiments, but also a 5 bp mismatch *puf-A* (5mmMO1) was employed as a negative control for MO1. Both *in vitro* and *in vivo* analyses were performed in Fig. S3 to examine the specificity for MOs. First, the addition of various amounts (0, 2, and 200 nM) of the *puf-A*-5mmMO1 did not affect the transcription/translation reactions for *puf-A in vitro*. Secondly, for *in vivo* experiment, the *puf-A* 5′-UTR and its partial coding region were added onto pEYFP-N1 plasmid which contained CMV promoter and YFP gene to generate the p*puf-A*-YFP plasmid. *In vivo*, approximately 31.2% of embryos injected with this p*puf-A*-YFP exhibited fluorescence at 100 pg/embryo dosage (Fig. S3B). However, co-injection with MO1 totally suppressed the YFP expression; in contrast, the mismatch control, 5mmMO1, did not affect the YFP expression (Fig. S3B). It is further noted that the phenotypes of morphants injected with 5mmMO1 were also normal at 3dpf, similar to WT zebrafish (Fig. S3C).

**Figure 4:**
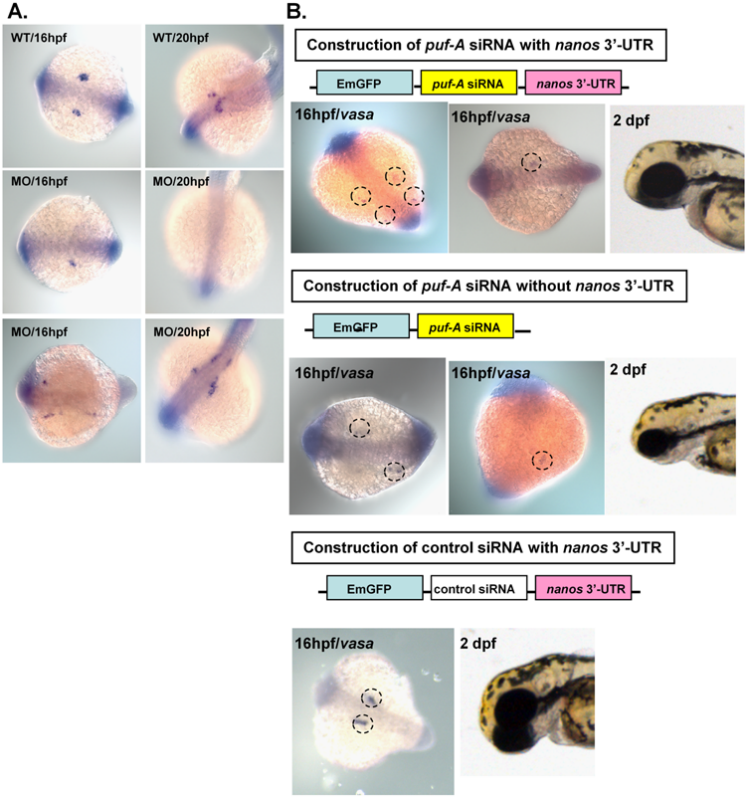
The *puf-A* gene plays a role in the PGC development in zebrafish embryonic development. (A) The *vasa* antisense riboprobe was used in whole-mount *in situ* hybridization as a marker to monitor primordial germ-cell (PGC) development in zebrafish. The *vasa* expression in wild-type (WT) and morphants (MO1 at 5 ng/embryo) at 16 and 20 hpf embryos in dorsal view. Anterior is to the left for 16 hpf and left bottom for 20 hpf. The morphants exhibited prominent abnormalities with either a reduction in PGC numbers (50.4%, n = 125 embryos) or abnormal patterns of migration (34.4%, n = 125) indicating the failure of PGC navigation towards their destined sites. (B) Upper: the construction of *puf-A* siRNA with *nanos* 3′ UTR, *vasa* expression and normal eye size in embryos 2 dpf after injection. The embryos displayed a marked reduction in PGC numbers (80.9%, n = 115 embryos) and abnormal patterns of migration (11.3%, n = 115 embryos). Middle: construction of *puf-A* siRNA without *nanos* 3′ UTR, *vasa* expression and small eye size in 2dpf embryos. Bottom: construction of control siRNA with *nanos* 3′ UTR, *vasa* expression and normal eye size in embryos after injection.

Furthermore, an independent approach using various siRNAs was performed in order to validate the MO data. The *puf-A* siRNA (i.e. without *nanos* 3′-UTR) was shown to suppress the zebrafish puf-A expression (Fig. S4) and generate a reduction in the eye size of zebrafish embryos after microinjection (i.e. compared with eye size when injected with control siRNA 2 dpf in Fig. 4B). Therefore, both MOs and siRNA knockdown analyses suggested that these genetic tools specifically knocked down the expression of *puf-A* leading to eye defects in zebrafish.

Several *in situ* markers, such as *emx3* (telencephalon marker), *krcx20* (rhombomere 3/5 marker), *pax6a* (forebrain, retina, hindbrain, spinal cord marker), *mab21l1* (retina, optic tectum and hindbrain marker), *mab21l2* (retina, optic tectum and hindbrain marker), *rx3* (retina marker), *six3b* (retina/diencephalon/midbrain marker) etc were used to characterize the eye defects during embryo development. As shown in Fig. S5A, *in situ* hybridization showed that most brain regions were normal at 1dpf, but some regions (like optic tectum and eyes) developed abnormal defects that occurred at 2dpf. For example, *six3b* expressed only in ganglion cell layer of eye tissues in wild type 2dpf; but this gene expressed all over in eye tissue in morphants (Fig. S5A). Furthermore, expression of another marker, *mab21l1*, was found in retina, optic tectum and hindbrain. In contrast, in morphants, no mab21ll expression was found in the entire eye tissue or optic tectum (Fig. S5A). Furthermore, as shown in Fig. S5B, puf-A knockdown promoted apoptosis in eye tissues at 1dpf as compared to control. It seemed that cell death occurred prior to retinal differentiation which occurred approximately 28–30 hpf [15]. Moreover, the other retinal differentiation markers (ath5/atoh7) were not expressed in morphants as late as 36 hpf in *in situ* experiments (pictures not shown), suggesting that the puf-A knockdown led to specific differentiation defects in eyes, not simply delayed development.

Subsequently, at the development stages of 3 and 5 dpf, eye sections of WT fish and morphants were further examined (Fig. 3E). In WT zebrafish, the retina comprises several layers of differentiated cells including retinal ganglion cells, the inner plexiform layer, amacrine cells, bipolars, outer plexiform layer, rods and cones, and pigmented cells. In contrast, morphants with *puf-A* gene knockdown exhibited features of an undifferentiated retina with loss of detailed architecture and a significant reduction in eye size. Structures such as the rod and cone layers were not concentrically organized and retinal ganglion cells and plexiform layers were not readily discernible (Fig. 3E).

### Defects of primordial germ-cell development in the MO and siRNA knockdown

During embryo development, primordial germ cells (PGC) follow a unique developmental path that is characterized by specification and migration of these cells to colonize the gonads where they differentiate into gametes. To investigate whether *puf-A* is involved in PGC development, the *puf-A* MO was used to knockdown its expression in the early stage of zebrafish embryos. *In situ* hybridization with zebrafish PGC-specific *vasa* RNA was employed as a marker [7], [16] to monitor PGCs migration (Fig. 4A). It has been reported that zebrafish PGC movement began with four random clusters before 6 hpf to form two clusters that would move to either side of embryo midline by the end of the first day of development [17]. As shown in Fig. 4 A, PGCs appeared in gonad regions as two clusters in WT embryos at the 16∼20 hpf stage. But the morphants exhibited prominent abnormalities at the same stage of development (Fig. 4 A) with either a reduction in PGC numbers (50.4%) or abnormal patterns of migration (34.4%) indicating the failure of PGC navigation towards their destined sites.

Since MO knockdown could affect various tissues in embryos, it remained unclear whether the abnormal patterns of PGC migrations were caused directly by specific knockdown of *puf-A* expression in PGCs. It was reported that the 3′ UTR of *nanos* plays critical roles in RNA stabilization and could assure specific expression of reporter gene in PGC regions [18], [19]. Herein, a *puf-A* siRNA prepared with *nanos* 3′ UTR was microinjected into embryos. These embryos showed normal size head and eyes up to 2 dpf but they already displayed a marked reduction in PGC numbers (80.9%) and abnormal patterns of migration (11.3%) at 16 hpf of development; (Fig. 4B). In contrast, the *puf-A* siRNA (without *nanos* 3′ UTR) had the similar phenotypes as MO morphants with small eyes, small head, and brain edema in addition to abnormal PGC migration and reduction in PGC number (Fig. 4B). Furthermore, the control siRNA with *nanos* 3′-UTR exhibited normal PGC migration and normal eye size development (Fig. 4B). Thus, specific knockdown of *puf-A* in PGCs led to abnormal PGC development in zebrafish embryo.

### Expression of the *puf-A* gene in eye tissues of adult mice

**Figure 5:**
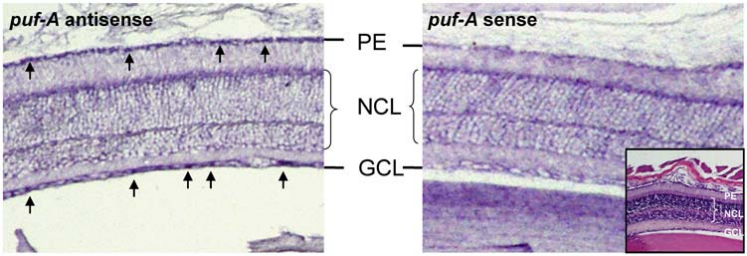
The *puf-A* gene was expressed in adult murine eyes. Panels show *in situ* hybridization of an adult mouse eye with NBT-BCIP color reaction after hybridization with puf-A antisense and sense probes, separately. The black arrows point to *puf-A* expression which was probed with antisense, while mouse sense probe served as the negative control. HE staining inside the mouse sense panel shows the different retinal layers of the mouse eye. PE, pigmented epithelium; NCL, nuclear cell layer.

The *puf-A* gene was identified in the mouse genome as the mouse *D19Bwg1357e* (Table 1). As *puf-A* MO knockdown led to abnormal differentiation in zebrafish eye, *puf-A* expression in mouse eyes was investigated. The *in situ* hybridization analysis showed that *puf-A* was expressed in retina ganglion cells of mice, and to a lesser degree, in the pigmented cells of mice as well (see arrows in Fig. 5), suggesting that the Puf-A protein may play an important role in the function of vertebrate eyes.

### Identification of the mRNA targets for puf-A in zebrafish

The biotinylated puf-A was prepared from *in vitro* transcription/translation) and then purified through immobilization on streptavidin magnetic beads. Afterwards, the purified biotinylated puf-A were mixed with 10 ug mRNA mixtures from embryos and ovaries. After fix and PBS wash, the residual RNA pulled down by biotinylated Puf-A was amplified, subcloned and sequenced. Using this pull-down assay, many potential RNA targets for puf-A bindings were found and listed in Table 2 with their gene IDs and symbols. We further showed that there was a reciprocal relationship for the expression of puf-A and one of its potential RNA targets, prdm1a. (Jui-Chin Chang and John Yu, unpublished observations). Therefore, these results and computer modeling predicted that puf-A is a RNA binding protein.

**Table 2:** The potential target RNAs identified from pull down assay with biotinylated puf-A*.

Gene IDs	Gene symbols	Names or descriptions	Locations (chromosome)
792333	zgc:193933	ovary-expressed homeobox protein	24
323473	prdm1a	PR domain containing 1, with ZNF domain	16
568830	spata2	spermatogenesis associated 2	23
327196	tex10	testis expressed 10	16
321726	rbb4	retinoblastoma binding protein 4	19
566947	ddx3	DEAD (Asp-Glu-Ala-Asp) box polypeptide 3	9
114438	zp2.2	zona pellucida glycoprotein 2.2	20

* detailed description of this pull-down assay was described in Text and Methods and Materials.

## Discussion

The zebrafish has become one of the top vertebrate models for genetic and developmental studies because it is highly prolific and amenable to micromanipulation and gene knockdown. In this study, the zebrafish was used as a model for analyzing the structure and functions of a novel gene, *puf-A*. This has proved to be an efficient strategy for a detailed analysis of the function of a gene. This approach provides an outstanding platform for understanding the functions of novel genes and their roles in controlling development of an organ or organism.

It was found that the *puf-A* gene was primarily expressed in the eyes and ovaries and to a lesser degree in the brain and kidneys of adult zebrafish. In the eyes of adult zebrafish and mice, the Puf-A protein was mainly expressed in retina ganglion cells. During embryogenesis, the formation of retinal neurons follows a phylogenetically conserved order, and all six retinal neuron types are generated from common multipotent progenitors, with retinal ganglion cells being the first neurons to occur [20], [21]. In this study, zebrafish morphants of 3- and 5-dpf embryos showed incomplete differentiation patterns in the retina, suggesting that the Puf-A protein may have important roles in the development of retinal progenitors.

Additionally, during embryonic development, knockdown of the *puf-A* gene led to a reduction in the number of PGCs and their abnormal migration, suggesting that Puf-A is involved in the maintenance and migration of these primitive germ cells. The adult zebrafish ovary is a useful vertebrate model to study oocyte development and its regulation [22]. In this study, the expression of *puf-A* was predominantly in stage I follicles in adult ovaries and became undetectable in stage II and III follicles during subsequent oocyte development. It was noted that the most primitive germline stem cells, oogonia, were not readily distinguishable from stage I follicles. Thus, the transition of oogonia into stage I follicles was not investigated in this study. Taken together, these findings indicated that in zebrafish Puf-A not only regulates PGC development but may also play a role in germline stem cells up to stage I follicles.

In total, 14 puf-related proteins of zebrafish, mice, and humans were identified by the SMART server. The Puf-A in this study with its newly identified roles in eyes and PGCs corresponds to the zebrafish LOC394185, mouse D19Bwg1357e, and human KIAA0020. There are three groups of Puf-related proteins: the Puf-A homolog cluster, the C14orf21 homolog cluster, and the PUM1/PUM2 homolog cluster. The Puf-A and C14orf21 homologs could be separated into two homolog clusters and the proteins of their members were similar to each other within the same cluster, but easily distinguishable from members of the other clusters. For example, the Puf-As in humans, mice, or zebrafish were similar to each other, but showed only ∼21% identity with the human Pumilio (PUM1) protein. In the PUM1/PUM2 homolog cluster, there are more than one member proteins in each animal species, especially in zebrafish (six homologous genes in the PUM1/PUM2 cluster have been annotated, but await further characterizations of their proteins).

The Puf family proteins are characterized by their tandem Puf repeats with ∼35–39 amino acids in each repeat. Each repeat consists of three α-helixes, which bind to its RNA recognition residues [8]. A typical RNA recognition motif such as the Pumilio homolog domain usually contains eight tandem Puf repeats. Our computer modeling indicated that the Puf domain of the Puf-A homologs in humans, mice, zebrafish and yeast (data not shown) consists of six Puf repeats and the topographic characteristics predicted it to be a new RNA binding protein. Furthermore, using a pull-down assay, we had found potential RNA targets for puf-A bindings, conceivably leading to the suppression of target gene expression. In fact, Puf6p, the homolog protein of puf-A in yeast, was shown to be involved in the repression of ASH1 mRNA [23]. This model of human Puf-A also suggested that its Puf domain exhibited a structural feature with six Puf-repeats and a middle region of the Puf domain that mimics exactly two additional Puf repeats. In addition, the asymmetric distribution of the electrostatic potentials of the amino acid side chains on the surfaces of the Puf domain of Puf-A suggests that the concave surface of the domain binds RNA, while the convex face may react with not yet identified interacting proteins such as nanos.

RNA-binding proteins play important roles in RNA-related cellular processes, including RNA splicing, export, translation, stabilization, and degradation [24], [25], [26]. The specific RNAs bound to Puf-A are being delineated. On the other hand, the homologs of *puf-A* in *Drosophila* and *C. elegans* have been reported to be *penguin* and *puf-12*, respectively. The function of the penguin protein in Drosophila is still unknown, while knockdown of *puf-12* via RNAi in the *C. elegans* caused early larval arrest and egg laying abnormalities (Egl) (http://www.wormbase.org/).

## Materials and Methods

### Animals

Breeding and maintenance of AB strain zebrafish, as well as collecting and staging of embryos, were done according to standard procedures [27]. Some embryos were reared in egg water treated with 0.003% 1-phenyl-2-thiourea (PTU) to inhibit pigmentation [27]. Developmental times refer to hours (hpf) or days (dpf) post-fertilization.

### RT-PCR and cDNA cloning of puf-A from zebrafish

Total RNA was extracted from zebrafish embryos and adult tissues using Tri-reagent (Sigma, St. Louis, MO, USA). Reverse transcription was performed using the Superscript pre-amplification system (Gibco BRL, Grand Island, NY, USA) as described in the manufacturer's instructions. The cDNA product was amplified by PCR with specific primer sets for *puf-A* or *β-actin*. The *puf-A* forward primer was 5′-GTTCAACAGAAAGCCGACAG-3′ and the reversed primer, 5′-CCAACATCACTTCACCTACC-3′. The *β-actin* forward primer was 5′- TCACACCTTCTACAACGAGCTGCG-3′ and the reversed primer, 5′- GAAGCTGTAGCCTCTCTCGGTCAG-3′. To obtain the *puf-A* complete cDNA, rapid amplification of 5′- and 3′-cDNA ends (5′-RACE and 3′-RACE) was performed with total RNA of the ovaries using the SMART cDNA amplification kit (Clontech Laboratories, Palo Alto, CA, USA). The RACE products were subcloned into pGEM-T easy vectors (Promega, Madison, WI, USA) and sequenced. The cDNAs of *puf-A* full-length and *puf-A* without 5′-UTR region were reconstructed into pBluescript SK minus vector using the 5′- and 3′-RACE products.

### Retrieval of putative Puf-protein sequences

Zebrafish, murine and human Puf protein sequences were retrieved from the SMART server (http://smart.embl-heidelberg.de) by the analysis of Pumilio-conserved domains in both the normal and genomic modes. Four individual sequences were found to contain the Puf domain in humans and mice. Additionally, there were six putative Puf-related proteins identified in zebrafish using the SMART server. More detailed information about these Puf proteins and their accession numbers is given in Data S1 and Table 1.

### Phylogenetic analysis

A multiple-sequence alignment for these Puf-related protein sequences was generated by CLUSTAL X2.0, using the BLOSUM series matrix [28]. The option for a negative matrix was turned on, while the other parameters remained at the default setting. The BLASTP algorithm with the BLOSUM62 matrix, which was implemented in BLAST at NCBI, was used for the sequence similarity analysis. A phylogenetic tree of putative Puf proteins was constructed using algorithms with PHYLIP vers. 3.67 [29] (see Data S1). The final unrooted tree diagram was prepared using DENDROSCOPE vers. 1.2.4 [30].

### Modeling the Puf domain of human Puf-A (KIAA0020)

In order to model the three-dimensional (3D) structure of human Puf-A, the mGenTHREADER method of the PSIPRED server [31], [32] was used for predicting secondary structures and making sequence alignments. Initially, the structural information of human Pumilio homolog domain (Protein Date Bank code: 1IB2 and 1M8Y) [8], [13] was used as modeling templates. Even though the sequences of human Pumilio (PUM1) is only 21% identity with human Puf-A, the crystal structure of Pumilio homolog domain is similar to Puf-A detected by mGenTHREADER with *p* value<0.0001.

Then 3D structure of Puf domain in human Puf-A was constructed by MODELLER 9v3 [33] (see Data S1). A segment of the RNA ligand from 1M8Y [13] was assembled into the resulting model to represent the potential RNA binding site. Furthermore, the electrostatic potentials were calculated using DELPHI [34] with default parameters setting in CHIMERA [35]. The color spectrum mapped onto the domain surface ranged from −7 kT/e (dark red) to +7 kT/e (dark blue). Finally, 3D structural diagrams in this study were prepared using CHIMERA [35]. The convex and concave surfaces represent the presentation of the model that had been rotated 180° about the vertical axis.

### In situ hybridization of eyes and ovaries

The collection and staging of embryos were performed as described. Embryos were fixed overnight at 4°C in 4% paraformaldehyde buffered with 1× phosphate-buffered saline (PFA/PBS). In addition, the ovaries and eyes were removed from zebrafish or mouse after anesthetization and decapitation, and placed in 4% PFA/PBS. After being treated with 30% sucrose, specimens were embedded in OCT. Frozen sections (7 and 10 µm thick for mouse and zebrafish, separately) were collected onto coated slides. *In situ* hybridization was performed using an InsituPro automated system (Intavis, Koeln, Germany). Whole-mount and section *in situ* hybridization were carried out using a digoxigenin (DIG)-labeled RNA probe and anti-DIG antibody conjugated with alkaline phosphatase as described previously [36], [37]. After hybridization, slides were incubated with anti-DIG antibody conjugated with AP, and developed with NBT-BCIP reagents. The *in situ* hybridization analysis of the cryosections of adult zebrafish eyes was carried out with a zebrafish *puf-A* riboprobe after fluorescein (Flu) labeling. After hybridization, slides were incubated with anti-Flu-AP, and developed with FastRed reagents.

The following DIG-labeled RNA probes were prepared from linearized plasmids using the DIG RNA labeling kit (Roche, Basel, Switzerland): (1) an antisense probe of the *puf-A* gene prepared from KpnI-digested pBluescript SK^−^-*puf-A* (full-lengh, 2,053 bp) using T3 RNA polymerase, (2) a *puf-A* sense probe prepared from BamHI-digested pBluescript SK^−^-*puf-A* using T7 RNA polymerase, and (3) a *vasa* antisense probe prepared from XbaI-digested pBluescript SK^−^-*vasa* (a gift from Dr. Bon-chu Chung, Academia Sinica) with T7 RNA polymerase. Follicles at different stages of development were identified according to the different-sized diameters of the follicles [9], [10].

### Morpholino (MO) knockdown

Zebrafish embryos were obtained by natural mating and MO microinjection was performed at the stage of 1∼4 cells. The *puf-A*-MO1 antisense oligonucleotide 5′-AATGGACCATGTGTACAGACAAACA-3′ was designed to direct against the 5′ UTR of the *puf-A* gene, and the *puf-A*-MO2 antisense oligonucleotide was 5′-TTTACCCTCCATAATGGACCATGTG-3′ that directed against the 5′ UTR and part of coding region including ATG. The 5 bp mismatch MO1 as a negative control for MO (i.e. *puf-A* 5mmMO1) was 5′-AATcGACgATGTGTAgAcACAAAgA-3′. Embryos positioned in an agarose injection chamber were injected with 5∼10 ng of MO in 4.6 nl using a Narishige micromanipulator and needle holder (Narishige, Tokyo, Japan). For the experiment, eye size was determined by photographing lateral views of anesthetized larvae and was normalized to the average eye size of age-matched WT fish.

### In vitro transcription/translation

An *in vitro* transcription/translation assay was carried out with the TNT Quick coupled reticulocyte lysate system together with the Transcend™ biotinylated lysine-tRNA (Promega), according to the manufacturer's protocol. MOs were added to the complete TNT Quick Master Mix to final concentrations of 0.1 or 10 µM and incubated at 30°C for 90 min. One microliter of this reaction mixture was resolved on 10% SDS/PAGE, and biotin-labeled lysine residues were detected on Western blots via a streptavidin-alkaline phosphatase and visualized with NBT-BCIP reagents.

### Rescue experiment of morphants

Rescue experiments were performed by injecting the synthesized capped *puf-A* RNA with *puf-A*-MO1. The capped *puf-A* RNA that did not contain a 5′ UTR region was prepared from a pBluescript SK-plasmid after BamHI digestion using the mMessage mMachine kit (Ambion, Austin, TX, USA). For the rescue experiments, 200 pg of capped *puf-A* RNA was microinjected with *puf-A*-MO1 into zebrafish embryos, and the eye size was measured at 2 dpf.

### Paraffin embedding and sectioning of mouse

Eyes from mouse were collected and placed in 4% paraformaldehyde. Tissue sections (3 µm thick) from paraffin-embedded tissue blocks were placed on charged slides, deparaffinized in xylene, rehydrated through graded alcohol solutions and stained with hematoxylin and eosin (H&E).

### Puf-A silencing in the zebrafish PGCs

To silence the *puf-A* expression in zebrafish with small interfering RNA (siRNA), the pcDNA6.2-GW/EmGFP-miR (Block-iT Pol II miR RNAi Expression Vector Kits, Invitrogen) was used to construct the *puf-A* siRNA plasmid according to the user manual. The region of nt1078 to 1098 for zebrafish *puf-A* was chosen for the engineered *puf-A* siRNA plasmid. The commercial pcDNA 6.2-GW/EmGFP-miR-neg control plasmid served as the “control siRNA”. In addition, the 3′ UTR fragment of *nanos* prepared from the PCR product of pGEM-T Easy-*nanos* plasmid (a gift from Dr. Bon-chu Chung) was subcloned into XhoI site of the *puf-A* siRNA and control siRNA plasmids, separately, to generate the plasmid with either *puf-A* siRNA or control siRNA containing *nanos* 3′ UTR. Therefore, there are four siRNA plamids: *puf-A* siRNA with or without *nanos* 3′ UTR and their two respective control plamids without *puf-A*.

Then, the PCR products generated from these four siRNA plasmids using forward primer (ACAAGTTTGTACAAAAAAGCAGGCT) and reverse primer (ACCACTTTGTACAAGAAAGCTGGGT), were subcloned into pGEM-T Easy vector using a TA cloning kit (Promega). Afterwards, using the mMessage mMachine kit (Ambion), the RNAs with *puf-A* containing either or no *nanos* 3′ UTR and their controls without *puf-A* were prepared separately. Finally, 100–200 pg of these *puf-A* siRNAs and control siRNAs (with or without *nanos* 3′ UTR) was microinjected into one-cell stage of zebrafish embryos, and the phenotypes and *vasa* expression were observed under microscope.

### Indentification of the mRNAs that are targets of puf-A in zebrafish

Briefly, biotinylated puf-A was prepared from *in vitro* transcription/translation kit using the TNT Quick coupled reticulocyte lysate system together with the Transcend™ biotinylated lysine-tRNA (Promega, Madison, WI, USA) and then purified through immobilization on streptavidin magnetic beads (Promega) with 5 times PBS wash. Afterwards, the purified biotinylated puf-A were mixed with 10ug mRNA mixtures from embryos and ovaries. After formaldehyde fix (final 1% concentration), glycine treatment (final 125mM concentration) and 5 times PBS wash, the residual RNA pulled down by biotinylated puf-A was amplified by using Full Spectrum Complete Transcriptome RNA Amplification kit (System Biosciences, Mountain View, CA, USA) as described in the manufacturer's instructions. The PCR products were subcloned into pGEM-T easy vectors (Promega) and sequenced.

## Supporting Information

Data S1 (0.05 MB DOC)

Figure S1 cDNA nucleotide sequence of the zebrafish puf-A gene. The full-length sequence of zebrafish puf-A cDNA was identified using 5′- and 3′-RACE. The 5′-untranslated region (UTR) and 3′-UTR are shown in lowercase letters and the coding region (nucleotides 45∼1924) in uppercase letters. The stop codon is marked with an *. The deduced amino acid sequence (629 amino acids) is shown below the nucleotide sequence. At residues 558∼562, the sequence is “Glu-Arg-Phe-Ser-Arg” in bold letters. Blue arrow indicates the location of MO1 target site; black arrow refers to the MO2 target site.
(8.20 MB TIF)

Figure S2 Multiple sequence alignments of Puf proteins. Sequences of 14 Puf proteins of human (Hs), mouse (Mm), and zebrafish (Dr) were aligned by CLUSTAL X as described in Methods. Protein names are shown at the left of the alignment data, and the residue numbers are shown at the right side. The quality scores of alignment are represented as column graph under the ruler to indicate the level of similarity among these proteins. The color scheme for the consensus residues was applied the default settings.
(9.43 MB TIF)

Figure S3 The in vitro and in vivo analyses for the specificity of puf-A MO1. (A) Various amounts of puf-A-5mmMO1 (5 bp mismatch control: 0, 2, and 200 nM) were added to the in vitro transcription/translation reactions for puf-A. One microliter of the reaction mixture was separated on 10% SDS/PAGE, blotted and incubated with streptavidin-AP, followed by development with NBT-BCIP reagents.(B) For in vivo experiment, the puf-A 5′-UTR and its partial coding region were added onto pEYFP-N1 plasmid which contained CMV promoter and YFP gene to generate ppuf-A-YFP. Then 4.6 ng of the puf-A-MO1 or puf-A-5mmMO1 was co-injected to embryo with ppuf-A-YFP plasmid at 100 pg per embryo. The numbers of embryo with YFP expression were enumerated at 1dpf. As shown, 15/48 embryos injected puf-A-YFP plasmid exhibited fluorescence at 100 pg/embryo dosage, while none of the 113 embryos co-injected with the puf-A-MO1 had YFP expression. In contrast, co-injection with the mismatch control, puf-A-5mmMO1, did not affect the expression of YFP. (C) The panels showed the phenotypes of WT and morphants at 3dpf after injection with 9.2 ng of puf-A-5mmMO1 dosage.
(7.79 MB TIF)

Figure S4 The puf-A siRNA suppressed specifically the zebrafish puf-A expression in 3T3 cell line. The 3T3 cell line was co-transfected with pFlag-puf-A and different siRNAs. In the first line of Western blot, the siRNA was the control siRNA (pcDNA 6.2-GW/EmGFP-miR -neg control plasmid) as negative control. In the middle line, ppuf-A siRNA and in the last line, ppuf-A siRNA containing nanos 3′-UTR were used to suppress the puf-A expression. Upper panel showed the Western blot after reaction with anti-Flag antibodies, while lower panel showed Western blot for β-actin as internal control.
(7.66 MB TIF)

Figure S5 Expression of in situ marker genes and elevated level of apoptosis in puf-A morphants (A) The upper four panels showed normal expression of krox20 and emx3 in WT and morphants, separately, at 1dpf. The lower four panels showed the abnormal expression patterns of six3b and mab21l1 in WT and morphants at 2dpf. (B) Apoptotic cells were detected by terminal deoxynucleotidyl transferase-mediated dUTP nick end labeling (TUNEL) using an In Situ Cell Death Detection kit (Roche). Embryos were fixed with 4% PFA and whole eyes at 1dpf were dissected out. Black arrows refer to the apoptotic cells in eyes of WT embryos. Acridine orange (AO) was also used to label apoptotic cells in zebrafish embryos. The average number of AO positive cells per retina in wild-type (WT, n = 8) and puf-A morphants (MO1, n = 8) at 1dpf was presented.
(9.88 MB TIF)
