# Supplementary material for: A Novel puf-A Gene Predicted from Evolutionary Analysis Is Involved in the Development of Eyes and Primordial Germ-Cells
Source: PLoS One. 2009 Mar 25;4(3):e4980. doi: 10.1371/journal.pone.0004980 (PMC2656619; doi:10.1371/journal.pone.0004980)
Supplement: Data S1 — (0.05 MB DOC) [file pone.0004980.s001.doc]

**Supplementary data**

*Accession numbers for Puf proteins*

Human and mouse Puf proteins were manually annotated in Swiss-Prot ([http://www.expasy.org](http://www.expasy.org/)) and the sequences in the Swiss-Prot were retrieved. The Swiss-Prot accession numbers for the human Puf proteins entries are KIAA0020 (Q15397) (Puf-A), C14orf21 (Q86U38), Pumilio PUM1 (Q14671), and PUM2 (Q8TB72). The murine Puf entries are D19Bwg1357e (Q8BKS9) (Puf-A), 2610027L16Rik (Q8BMC4), Pumilio PUM1 (Q80U78), and PUM2 (Q80U58). The six Puf proteins of zebrafish, however, were retrieved from the Reference Sequence (RefSeq) database at NCBI (http://www.ncbi.nlm.nih.gov). Their accession numbers are Puf-A (XP_695580.2), LOC564287 (XP_692728.2), LOC568777 (XP_697221.2), LOC567494 (NP­_001096040.1), LOC569578 (XP_698067.2), and LOC798171 (XP_001338629.1).

*Phylogenetic analysis by PHYLIP vers. 3.67*

Generally, the phylogenetic analysis was done with standard procedure and default settings defined in PHYLIP vers. 3.67 [1].Bootstrapping was performed by SEQBOOT with 1,000 replicates for the PAM substitution model. PROTDIST was then used to calculate distance matrices of sequences in multiple datasets with the Jones-Taylor-Thornton model. The analysis of distance matrices with FITCH generated phylogenies using the Fitch-Margoliash method. Subsequently, CONSENSE was applied to construct the consensus tree by the majority-rule method. Additionally, the branch lengths were calculated by PROTDIST and FITCH according to the original sequence alignment generated by CLUSTAL X [2]

*Homology Modeling by MODELLER 9v3*

Then 3D structure of Puf domain in human Puf-A was constructed by MODELLER 9v3 [3] using functions of the AUTOMODEL class in python scripts with multiple-template mode. A segment of the RNA ligand from 1M8Y [4] was assembled into the resulting model to represent the potential RNA binding site. The refine level of molecular dynamics and simulated annealing was set to "refine.very_slow", and the Discrete Optimized Protein Energy (DOPE) method [3] was used to select the best model from the 50 initially generated models. The loop regions of the selected model were then refined iteratively by functions of the LOOPMODEL class. Finally, the refined model was subjected to energy minimization further by DEEPVIEW v3.7 using the GROMOS 43B1 force field till the delta E between two steps below 0.05 KJ/mol [5]. In addition to the DOPE method, the quality of the homology model was evaluated by the VADAR server (http://redpoll.pharmacy.ualberta.ca/vadar/), a web server for quantitative evaluation of protein structure quality [6].

*Similarity analyses of zebrafish Puf proteins*

Protein LOC568777 was slightly more similar to human Pumilio (PUM1) than to PUM2, sharing 92% identity in the aligned 284 residues. However, its gene description (LOC568777) in the Entrez Gene database was annotated as being similar to PUM2. In contrast, protein LOC567494 was somewhat more similar to human PUM2 than to Pumilio (PUM1), sharing 78% identity in the aligned 398 residues. Furthermore, protein LOC569578 contained only 164 amino acids and was more similar to human PUM2 than to PUM1. They shared 87% identity in the aligned 154 residues. The other protein LOC798171, consisting of only 182 amino acids, shared 89% identity in the aligned 117 residues with human Pumilio (PUM1), and similarly, shared 88% identity in the aligned 117 residues with human PUM2.

**Literature cited**

1. Felsenstein J (2007) PHYLIP (Phylogeny Inference Package) version 3.67. version 3.67 ed. Seattle: Department of Genome Sciences, University of Washington.

2. Larkin MA, Blackshields G, Brown NP, Chenna R, McGettigan PA, et al. (2007) Clustal W and Clustal X version 2.0. Bioinformatics 23: 2947-2948.

3. Eswar N, Webb B, Marti-Renom MA, Madhusudhan MS, Eramian D, et al. (2006) Comparative protein structure modeling using Modeller. Curr Protoc Bioinformatics Chapter 5: Unit 5 6.

4. Wang X, Zamore PD, Hall TM (2001) Crystal structure of a Pumilio homology domain. Mol Cell 7: 855-865.

5. Guex N, Peitsch MC (1997) SWISS-MODEL and the Swiss-PdbViewer: an environment for comparative protein modeling. Electrophoresis 18: 2714-2723.

6. Willard L, Ranjan A, Zhang H, Monzavi H, Boyko RF, et al. (2003) VADAR: a web server for quantitative evaluation of protein structure quality. Nucleic Acids Res 31: 3316-3319.
