# Supplementary figures and images for: A Novel puf-A Gene Predicted from Evolutionary Analysis Is Involved in the Development of Eyes and Primordial Germ-Cells
Source: PLoS One. 2009 Mar 25;4(3):e4980. doi: 10.1371/journal.pone.0004980 (PMC2656619; doi:10.1371/journal.pone.0004980)

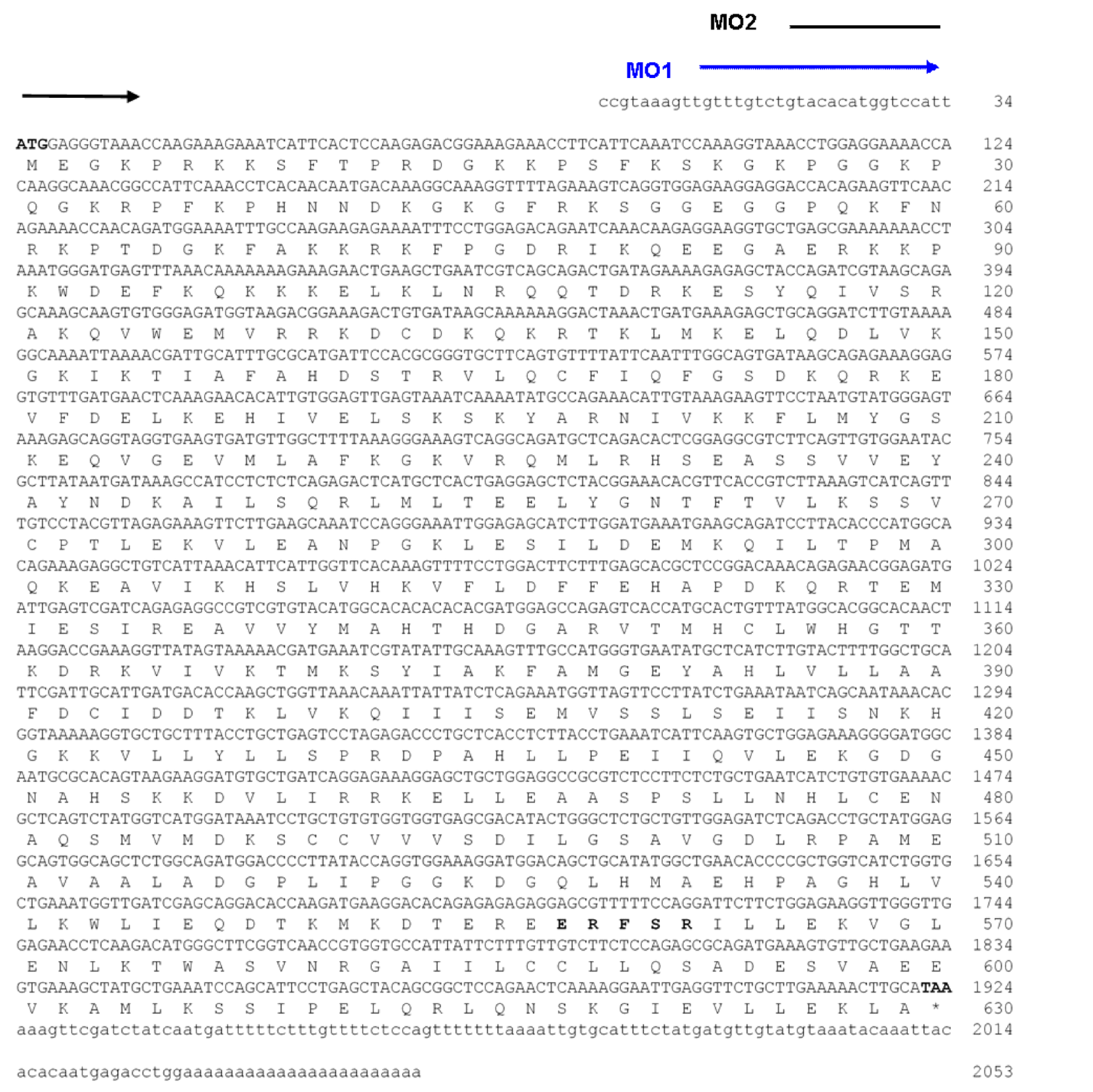

Supplement: Figure S1 — cDNA nucleotide sequence of the zebrafish puf-A gene. The full-length sequence of zebrafish puf-A cDNA was identified using 5′- and 3′-RACE. The 5′-untranslated region (UTR) and 3′-UTR are shown in lowercase letters and the coding region (nucleotides 45∼1924) in uppercase letters. The stop codon is marked with an *. The deduced amino acid sequence (629 amino acids) is shown below the nucleotide sequence. At residues 558∼562, the sequence is “Glu-Arg-Phe-Ser-Arg” in bold letters. Blue arrow indicates the location of MO1 target site; black arrow refers to the MO2 target site. (8.20 MB TIF) [file pone.0004980.s002.tif]

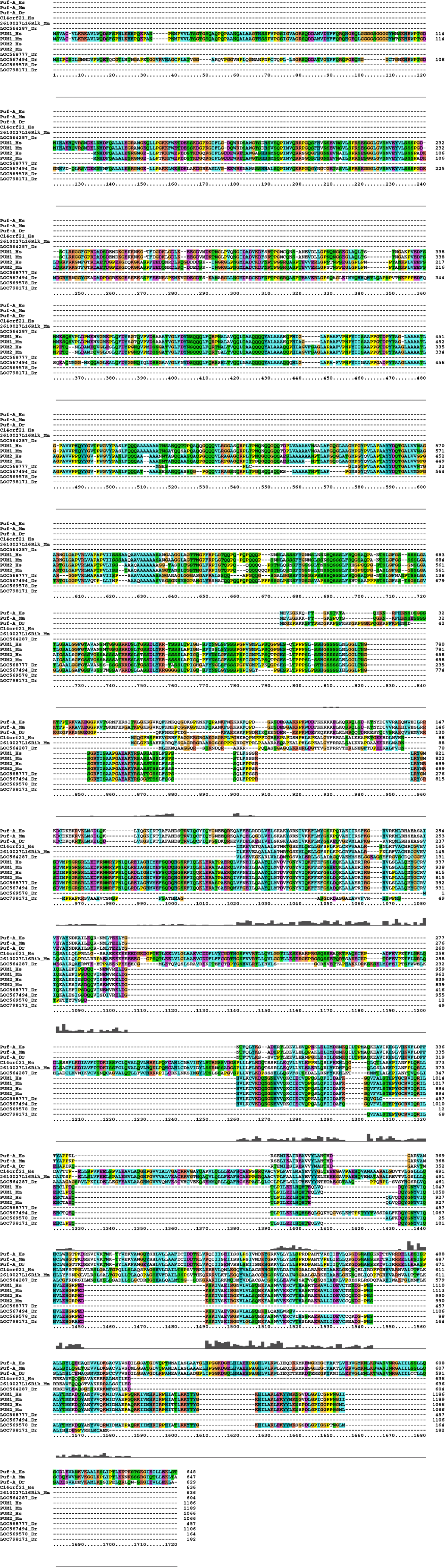

Supplement: Figure S2 — Multiple sequence alignments of Puf proteins. Sequences of 14 Puf proteins of human (Hs), mouse (Mm), and zebrafish (Dr) were aligned by CLUSTAL X as described in Methods. Protein names are shown at the left of the alignment data, and the residue numbers are shown at the right side. The quality scores of alignment are represented as column graph under the ruler to indicate the level of similarity among these proteins. The color scheme for the consensus residues was applied the default settings. (9.43 MB TIF) [file pone.0004980.s003.tif]

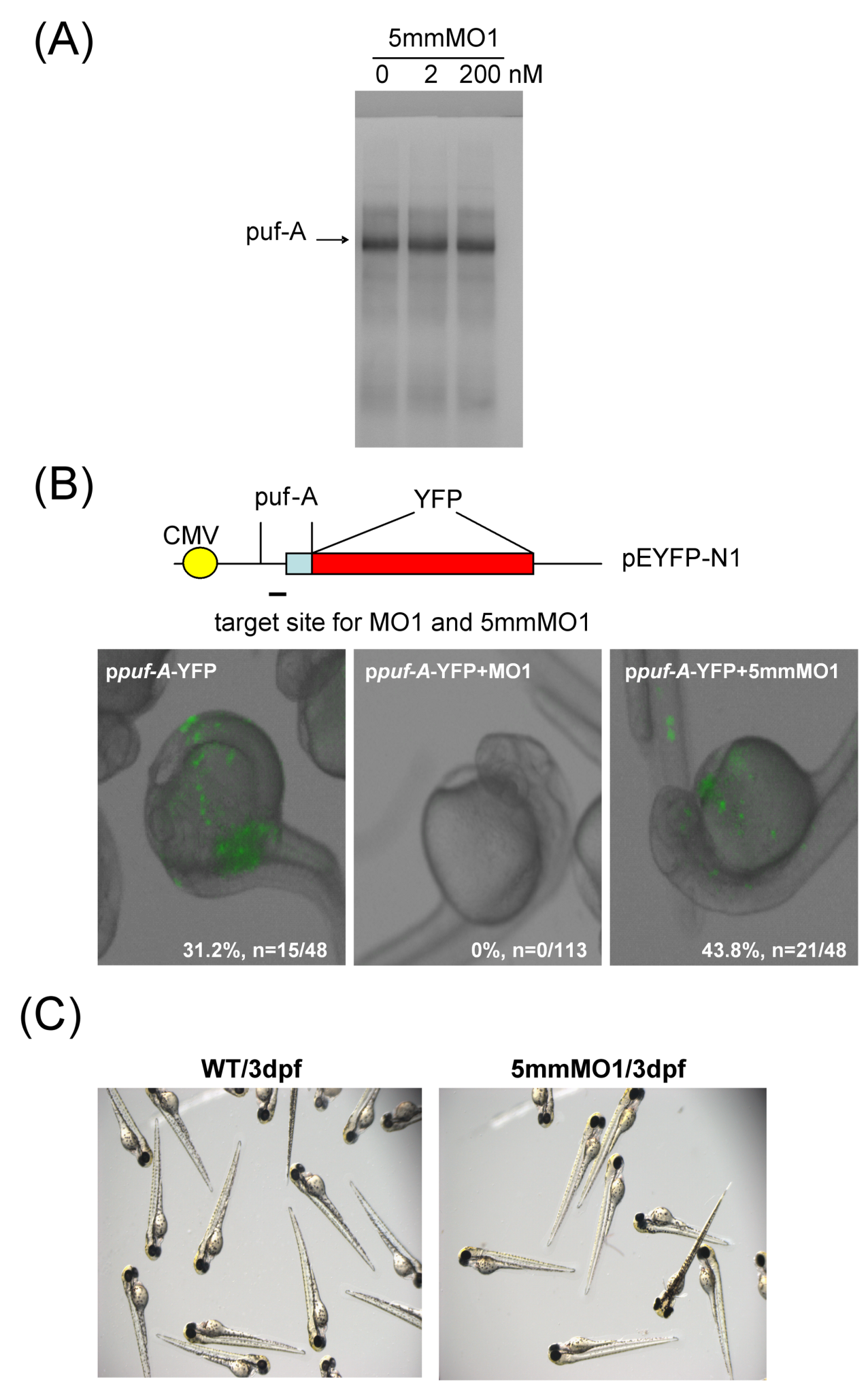

Supplement: Figure S3 — The in vitro and in vivo analyses for the specificity of puf-A MO1. (A) Various amounts of puf-A-5mmMO1 (5 bp mismatch control: 0, 2, and 200 nM) were added to the in vitro transcription/translation reactions for puf-A. One microliter of the reaction mixture was separated on 10% SDS/PAGE, blotted and incubated with streptavidin-AP, followed by development with NBT-BCIP reagents.(B) For in vivo experiment, the puf-A 5′-UTR and its partial coding region were added onto pEYFP-N1 plasmid which contained CMV promoter and YFP gene to generate ppuf-A-YFP. Then 4.6 ng of the puf-A-MO1 or puf-A-5mmMO1 was co-injected to embryo with ppuf-A-YFP plasmid at 100 pg per embryo. The numbers of embryo with YFP expression were enumerated at 1dpf. As shown, 15/48 embryos injected puf-A-YFP plasmid exhibited fluorescence at 100 pg/embryo dosage, while none of the 113 embryos co-injected with the puf-A-MO1 had YFP expression. In contrast, co-injection with the mismatch control, puf-A-5mmMO1, did not affect the expression of YFP. (C) The panels showed the phenotypes of WT and morphants at 3dpf after injection with 9.2 ng of puf-A-5mmMO1 dosage. (7.79 MB TIF) [file pone.0004980.s004.tif]

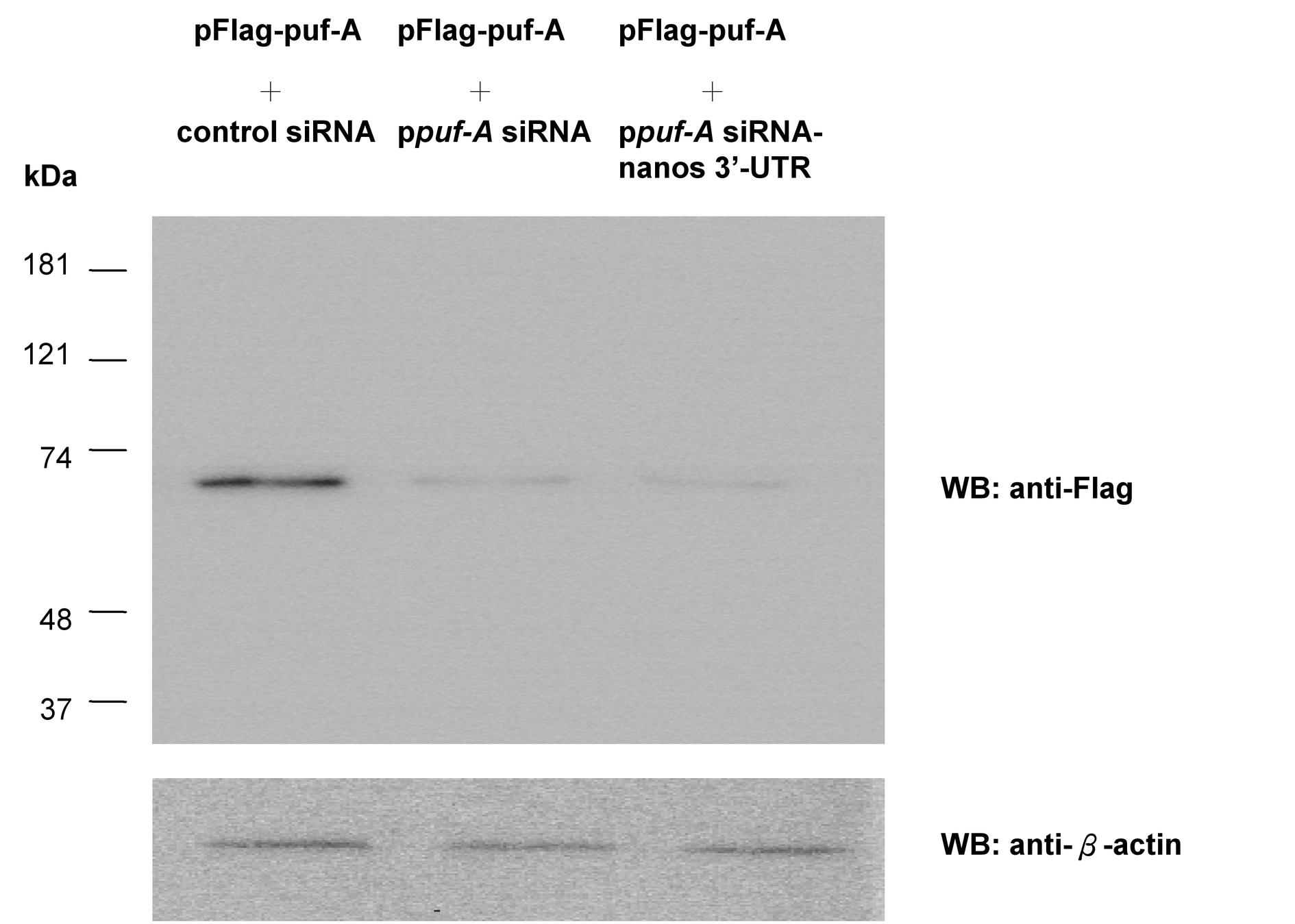

Supplement: Figure S4 — The puf-A siRNA suppressed specifically the zebrafish puf-A expression in 3T3 cell line. The 3T3 cell line was co-transfected with pFlag-puf-A and different siRNAs. In the first line of Western blot, the siRNA was the control siRNA (pcDNA 6.2-GW/EmGFP-miR -neg control plasmid) as negative control. In the middle line, ppuf-A siRNA and in the last line, ppuf-A siRNA containing nanos 3′-UTR were used to suppress the puf-A expression. Upper panel showed the Western blot after reaction with anti-Flag antibodies, while lower panel showed Western blot for β-actin as internal control. (7.66 MB TIF) [file pone.0004980.s005.tif]

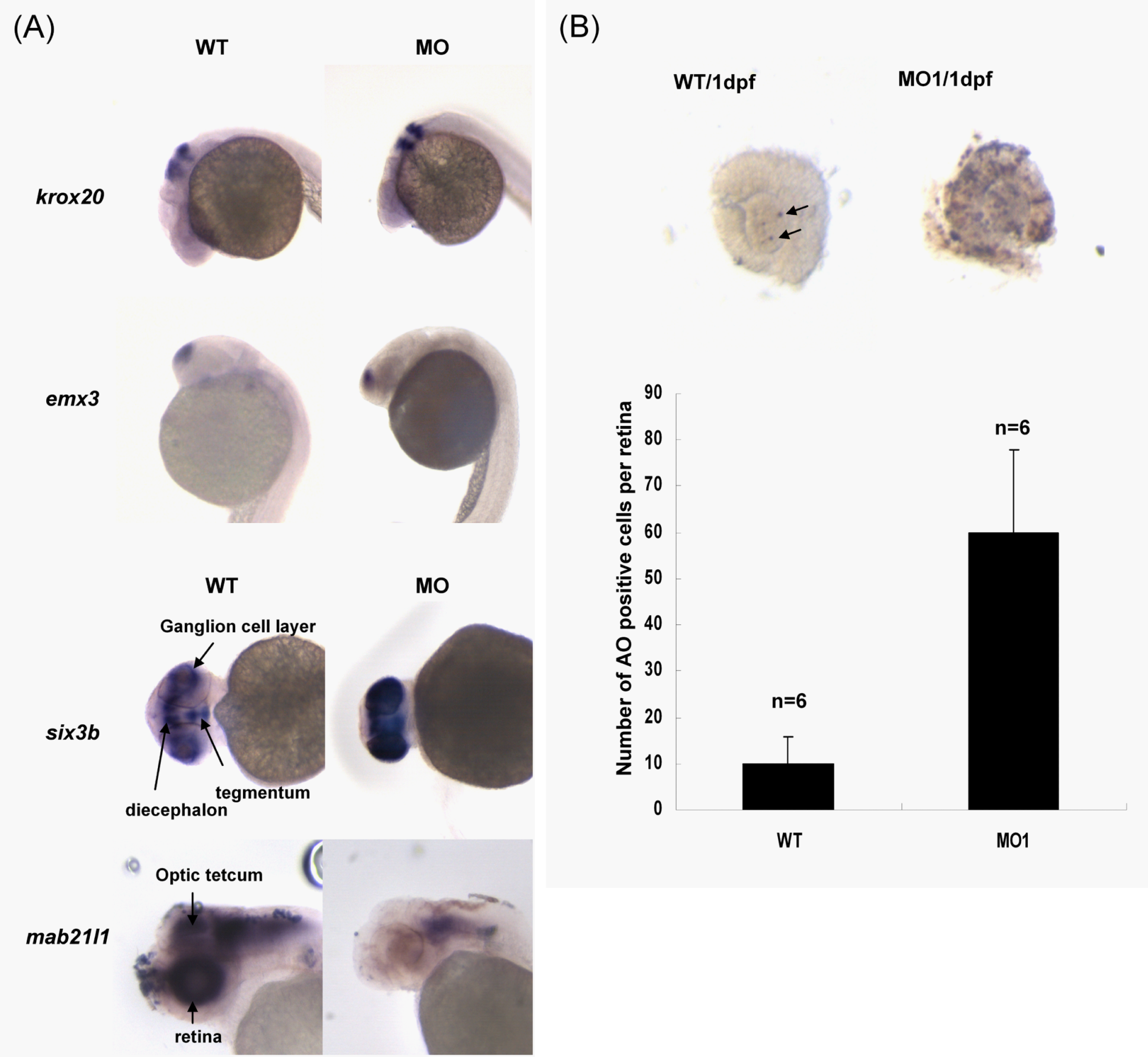

Supplement: Figure S5 — Expression of in situ marker genes and elevated level of apoptosis in puf-A morphants (A) The upper four panels showed normal expression of krox20 and emx3 in WT and morphants, separately, at 1dpf. The lower four panels showed the abnormal expression patterns of six3b and mab21l1 in WT and morphants at 2dpf. (B) Apoptotic cells were detected by terminal deoxynucleotidyl transferase-mediated dUTP nick end labeling (TUNEL) using an In Situ Cell Death Detection kit (Roche). Embryos were fixed with 4% PFA and whole eyes at 1dpf were dissected out. Black arrows refer to the apoptotic cells in eyes of WT embryos. Acridine orange (AO) was also used to label apoptotic cells in zebrafish embryos. The average number of AO positive cells per retina in wild-type (WT, n = 8) and puf-A morphants (MO1, n = 8) at 1dpf was presented. (9.88 MB TIF) [file pone.0004980.s006.tif]
